# The mediating effect of self-perceived aging on social capital and depression among Chinese community-dwelling older adult: a cross-sectional study

**DOI:** 10.3389/fpubh.2025.1571977

**Published:** 2025-06-25

**Authors:** Yuguo Ye, Hui Liu, Ziming Wang, Qianwei Zhang, Shifan Yang, Lian Yang

**Affiliations:** ^1^Faculty of Public Health & Policy, London School of Hygiene & Tropical Medicine, London, United Kingdom; ^2^Sichuan Development Centre for Healthy Aging, Chengdu, China; ^3^China Center for Health Development Studies, Peking University, Beijing, China; ^4^Department of Nutrition and Food Hygiene, West China School of Public Health and West China Fourth Hospital, Sichuan University, Chengdu, China; ^5^HEOA Group, School of Public Health, Chengdu University of Traditional Chinese Medicine, Chengdu, China

**Keywords:** social capital, depression, self-perceived aging, older adult, mediation analysis, cross-sectional study

## Abstract

**Background:**

Amid an aging society, boosting older adult social capital is crucial to meet their growing health needs. This could be an effective way to alleviate the shortage of public health resources and improve the mental health of the older adult. However, the underlying mediation pathways of how social capital affects the mental health of the older adult are not yet clear. This study aims to explore whether social capital has an impact on the depression levels of community-dwelling older adult and whether self-perceived aging mediates the relationship between social capital and depression, while providing a theoretical basis for scientifically constructing mental health intervention programs for the older adult.

**Method:**

A cross-sectional study was conducted from June to December 2022 in Chengdu, China. A stratified sampling survey of 1809 community-dwelling older adult individuals was conducted. Data on sociodemographic characteristics, social capital, self-perceived aging, and depression were collected. Univariate analysis was used to compare the depression differences among community-dwelling older adult with different sociodemographic characteristics. Pearson correlation analysis was used to explore the correlations between social capital, self-perceived aging, and depression. The SPSS PROCESS macro program was used to test the mediating effect of self-perceived aging between social capital and depression.

**Results:**

The mean score of depression was 39.07 (SD 13.97). Univariate analysis showed that there were statistically significant differences in depression scores among community-dwelling older adult of different age, marital status, chronic disease, medical insurance, endowment insurance, and monthly income per capita (*p* < 0.05). Social capital is negatively correlated with self-perceived aging (*r* = −0.418, *p* < 0.001) and also negatively correlated with depression (*r* = −0.263, *p* < 0.001), while self-perceived aging is positively correlated with depression (*r* = 0.324, *p* < 0.001). Social capital was negatively correlated with depression (*β* = −0.477, *p* < 0.001), and self-perceived aging partially mediated the relationship between social capital and depression, with a mediating effect of −0.180 (95% bootstrap CI −0.225 ~ −0.139), accounting for 37.7% of the total effect.

**Conclusion:**

Self-perceived aging played a partial mediating role between social capital and depression. It is recommended that relevant management agencies, communities, and families take effective measures to enhance the social capital of the older adult, help them build a positive self-perceived aging, and thereby reduce the risk of depression.

## 1 Introduction

Aging in China is a particularly pressing issue, given that the country has already transitioned into an aging society. As this demographic trend persists, it will further intensify the burden on both family and public healthcare systems (1). The aging trend is sign significant with the population of individuals aged 60 and above exceeding 297 million, accounting for 21.1% of the total population (2). In the face of such a large older adult population, their mental health issues have become a focus of concern for the nation and society. Depression has become a common mental disorder affecting the older adult in China (3, 4). According to the fourth round of the China Health and Retirement Longitudinal Study (CHARLS) in 2018, approximately 50.6% of older adult individuals in both urban and rural areas exhibit symptoms of depression (5). This grim reality urgently demands that we identify and validate modifiable risk factors and effective intervention measures. In the last decade, the Chinese government has given high priority to the mental health and social integration of the older adult, actively enhancing relevant policies and services (6). Against this backdrop, social capital, as an important social resource, has gradually attracted widespread academic attention for its potential impact on the mental health of the older adult.

Social capital has been defined in diverse ways across the literature (7). Over time, scholarly discourse has extended its concept from an individual asset to a feature of communities and even nations (8). Robison et al. (9) argued that definitions of social capital should be limited to what social capital is, rather than including statements about its applications, such as where it resides or what is can be used to achieve. In our study, social capital is defined as a person’s or group’s sympathy toward another person or group that may produce a potential benefit, advantage, and preferential treatment for another person or group of persons beyond that expected in an exchange relationship (9).

Measurement is a critical topic for social capital research. There is no consensus on social capital measurements, and different constructs of social capital are usually overlapping (10, 11). This study identified two validated Chinese-language social capital scales that are widely cited in the literature (12, 13). Based on an international multidisciplinary literature review, Gui and Huang developed a Chinese-language multidimensional scale to measure community social capital, incorporating seven key dimensions: local networks, sentiment, cohesion, non-local sociability, volunteerism, reciprocity and general trust, and community trust. Their analysis of data from 50 communities demonstrated strong reliability and validity. Subsequent English-language cross-sectional social capital studies have successfully applied and adapted this multidimensional scale (14, 15). Chen et al. (12) developed another validated social capital measurement index specifically for the Chinese context, also in Chinese language, employing a rigorous methodology that combined a literature review, expert panel discussions, and a two-round Delphi study involving 34 field experts to ensure robust reliability and validity. The eventually established index system included 5 first (including individual, family, community, workspace, and macro social capital), 23 s, and 50 third level indicators. Subsequent English-language cross-sectional studies adopted and adapted this multi-level social capital scale (16, 17).

Research shows that social capital not only provides individuals with emotional support, information exchange, and practical assistance but also promotes community cohesion, thereby helping to combat psychological distress, including depressive mood (18). Social capital is particularly important in the role of depression because it is directly related to an individual’s sense of social integration and belonging (19), which are crucial cornerstones of mental health. When individuals possess greater social capital, they are more likely to receive emotional support from friends and family, a vital resource in alleviating stress and combating depression (20, 21). However, despite studies indicating a positive correlation between social capital and mental health in the older adult (22–25), there is still a lack of in-depth discussion on how it specifically affects older adult depression, especially from the perspective of self-perceived aging (SPA).

Self-perceived aging, which refers to an individual’s subjective feelings and cognition about their own aging process (26, 27), is one of the key factors affecting the mental health of the older adult. Individuals with high SPA often hold more negative attitudes towards aging, and this negative cognition may exacerbate feelings of loneliness and helplessness, thereby increasing the risk of depression (28, 29).

Research indicates that social capital shapes SPA through multiple levels of influence. At the individual, family, and community social capital levels, Li et al. (30) found that marital status, frequency of children’s visits, and self-reported health indirectly affect SPA by reducing loneliness, while time spent outdoors indirectly influences SPA through improvements in daily living activities and social connectedness. According to Liu et al. (31), individuals more involved in grandchild care reported feeling older at younger ages. Additionally, workplace social capital also plays a critical role, as Tybjerg-Jeppesen et al. (32) demonstrated that a positive intergenerational work climate enhances SPA, increases work engagement, and reduces turnover intention. Liu et al. (31) found that individuals involved in political or community activities reported stronger perceptions of aging than their non-participating counterparts. At the society social capital level, Xiao et al. (33) identified the forms of healthcare and harmonious social environment as key determinants of SPA among older adults. Therefore, we hypothesize that self-perceived aging may be an important mediating variable between social capital and older adult depression. This hypothesis is supported by the study which indicates that SPA can significantly impact the mental health outcomes of the older adult (34). Ultimately, our study’s hypothesis is grounded in the following logical framework: individuals or groups with greater social capital (i.e., those who enjoy exchanges of relational goods and have rich sympathy toward another person or group that may produce a potential benefit, advantage, and preferential treatment for another person or group of persons beyond that expected in in exchange relationships across individual, family, community, association, and society levels) are more likely to form a more positive SPA, thereby reducing the risk of depression. SPA is not only a core variable influencing the mental health of older adults, directly related to their sense of happiness and satisfaction in later life, but also a malleable factor that can be significantly improved through psychological interventions and social support. Therefore, by exploring the mediating role of SPA in the relationship between social capital and older adult depression, we can not only gain a deeper understanding of the causes of older adult depression but also provide a scientific basis for developing more precise and effective mental health intervention strategies.

Communities, as the primary venues for the lives and social interactions of the older adult, offer unique advantages for mental health interventions (35), such as easy access, feasibility of implementation, and cost-effectiveness (36). However, community-dwelling older adult individuals not only face the gradual decline of physical functions (37), but also bear multiple pressures such as changes in social roles and family structures, all of which may affect their mental health status (38). Moreover, the failure to correctly distinguish between depression and normal aging, insufficient societal attention to the mental health of the older adult, and social prejudice against individuals with depression lead to low recognition and inadequate treatment of older adult depression (39, 40). As the aging population intensifies, these issues will become more pronounced.

**Figure 1:**
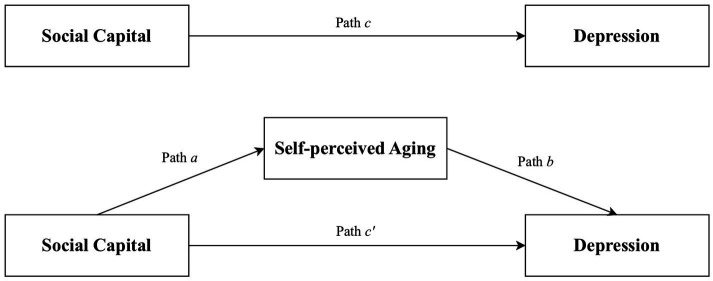
Proposed models that investigate mediated effects in the association between social capital and depression.

In this context, this study centers on community-dwelling older adult in China, aiming to explore the relationship between social capital and depression of older adults and factors that mediate the association. By delving into these relationships, we hope to provide a scientific basis for developing more precise and effective mental health intervention strategies for the older adult. We developed the following priori hypotheses (Figure 1): (1) Social capital will be negatively correlated with depression; (2) Self-perceived aging will mediate the relationship between social capital and depression.

## 2 Methods

### 2.1 Study population and data collection

This is a cross-sectional study conducted in the main urban districts of Chengdu City, Sichuan Province, China, from June to December 2022. Chengdu was chosen as the research location not only because of its leading economic position in Western China, high urbanization rate, and strong population appeal, but also due to its higher aging population proportion compared to the national average. This characteristic makes Chengdu an ideal place to study aging-related issues. A multi-stage cluster (stratified) random sampling method was employed. Firstly, based on the administrative division of Chengdu City, five central urban districts comprising only urban streets and communities were sampled. Secondly, two communities were randomly selected from each of the five urban districts, resulting in a total of 10 communities being chosen as the study’s sampling points. Finally, older adult individuals, in the selected communities with singular number of units and singular number of houses, who met the inclusion criteria were recruited to participate in a questionnaire survey.

Ten medical graduate students were recruited as interviewers and given them 1-week standardized training before data collection. The training covered research purposes, interview strategies and coding methods. The completed questionnaire and the coded entry process of the questionnaire were reviewed by two researchers daily during the survey period to ensure completeness of investigation and correctness of entry. The older adult filled out the questionnaire by themselves through face-to-face survey; for those who had difficulty in reading and writing, investigators would read questions for them and filled out questionnaires based on the answers of the older adult. Inclusion criteria: Permanent residents in main urban areas aged ≥ 60 years in Chengdu. Consciousness and able to express their current situation clearly. Exclusion criteria: Severe chronic non-infectious diseases based on International Classification of Diseases-10 (ICD-10), such as serious mental diseases, heart failure, renal failure, liver failure, malignant tumors. Severe mental diseases according the diagnostic and statistical manual of mental disorders-5(DSM-5), such as Schizophrenia, Bipolar disorder.

Sample size calculation was based on the formula: $n=\frac{\mu_{\alpha /2}^{2}\pi (1-\pi )}{\delta^{2}}$ . *δ* represents the margin of error, taken as 2.5%, *α* = 0.05, and $\mu_{\alpha /2}$ =1.96. Literature indicates that the prevalence of depressive symptoms among individuals aged 60 and above in China is 50.4%, hence *π* = 50.4%. The calculation yielded a required sample size of 1,537 for this study. Considering design effects, missing data, and non-response, to ensure a sufficient number of participants, the adjusted sample size was taken as 1.2 times the calculated sample size, resulting in a final sample size of approximately 1844 individuals. In the end, 1809 respondents completed the questionnaire, of which 1706 questionnaires were completed and valid, with an effective rate of 94.3%. This study was ethically approved by the Medical Ethics Committee of the Affiliated Hospital of Chengdu University of Chinese Medicine (approval no. 2023KL-011). The investigation was conducted in accordance with the Declaration of Helsinki.

### 2.2 Sociodemographic variables

The sociodemographic questionnaire was developed by the researchers, and the information included gender, age (60–69, 70–79 and ≥80 years), marital status (married and divorce/separate/widowed), education level (primary school or below, junior high school, senior high school, college or above), whether have chronic disease, whether have medical insurance, whether have endowment insurance and monthly income per capita (<1,000, 1,000–1,999, 2,000–2,999 and >3,000). Chronic diseases include hypertension, cardiovascular disease and diabetes mellitus. Endowment insurance also calls old age insurance, it is to point to national legislation compulsory collection of social insurance premium, and the formation of pension fund, pension is paid when the laborer retires, in order to ensure the social security system that its basic life needs (Supplementary Table S1).

### 2.3 Measurement of social capital

We adapted Chen et al.’s validated Chinese health-related social capital scale to develop our study-specific questionnaire (12). Due to the abstract nature of social capital as a construct (41), we operationalized it through proxy indicators reflecting its functional levels. We adapted a first-level indicator from “workplace” to “association” to more accurately capture retirees’ organizational engagements. Corresponding secondary indicators were also refined to align with our study purpose. The social capital scale in this study has 15 items, which are divided into five levels: individual, family, association, community, and society. The first level was individual social capital with 3 variables, measuring individual’s social network (e.g., you have lots of close relationship in your life). The second level was family social capital with 4 variables, measuring the relationship with family members and available support from family (e.g., your family is always around you). The third level was association dimension with 2 variables, including participation in organization activities (e.g., in the past year, you have usually participated in organization activities). The forth level was community social capital with 3 variables, including trust neighborhood and sense of belonging (e.g., if I have to move out of my present place, you will feel reluctant to do so). The last level was society dimension with 3 variables, concerning trust and equity (e.g., you have a lot of trust in medical institutions like hospitals and CDC). The answers consisted of 5-point Likert scales. The response categories were: 1 = strongly disagree to 5 = strongly agree. The total score was 15–75 points. The higher the score of each dimension and the overall total score, the higher the level of social capital. The Cronbach’s *α* value of the health-related social capital scale was 0.711 (Supplementary Table S2).

### 2.4 Measurement of self-perceived aging

The B-APQ (Brief Aging Perceptions Questionnaire), developed by Sexton et al. (42) based on the Leventhal self-adjustment theory model, measured SPA (42, 43). This questionnaire comprises 21 items across five dimensions: timeline-chronic, consequences positive, consequences and control negative, control positive, and emotional representations. Responses are recorded using a 5-point Likert scale ranging from 1 = strongly disagree to 5 = strongly agree. These five dimensions can be categorized into two broader aspects: negative SPA and positive SPA (44). Negative SPA encompasses the dimensions of timeline-chronic, consequences and control negative, and emotional representations. In contrast, positive SPA is constituted by consequences positive and control positive. It is important to note that the scoring for positive SPA (consequences positive and control positive) is reversed. The total score was 21–105 points. The higher the score of each dimension and total score of SPA, the more negative the SPA. The reliability of the B-APQ scale is supported by a Cronbach’s *α* value of 0.821 (Supplementary Table S3).

### 2.5 Measurement of depression

In this study, the Self-rating Depression Scale (SDS) was utilized to assess the depressive symptoms among the community-dwelling older adult population. The SDS, developed by Zung et al. (45), consists of 20 items scored using a 4-point Likert scale (46, 47). Ten of these items, specifically 2, 5, 6, 11, 12, 14, 16, 17, 18, and 20, are reverse-scored. A lower score indicates a better mental state. For ease of comprehension and comparison, the raw SDS score is multiplied by 1.25 to obtain the standard score. The total score was 25–100 points. The Cronbach’s *α* value for the SDS scale is 0.963.

### 2.6 Statistical analysis

The purpose of this study was to explore the pathway of SPA and social capital’ effects on depression in the older adult. Therefore, the mediating effect of SPA was the main objective of data analysis in this study. Data analysis was conducted using SPSS 23.0 software. Continuous variables that were normally distributed, such as social capital, SPA, and depression, were expressed as mean ± standard deviation (SD). Categorical variables (such as age, gender, education, etc.) were presented as *N* (%). Independent samples t-tests and one-way ANOVA were employed to assess differences in SDS score (depression) among community-dwelling older adult with varying sociodemographic characteristics. Pearson correlation analysis was utilized to investigate the correlations between social capital, SPA, and depression.

The hypothesized mediation model (Figure 1) was tested using the PROCESS macro in SPSS, a widely recognized tool for examining mediating effects in current research. Social capital was designated as the prediction variable, SPA as the mediator, and depression as the outcome variable. Sociodemographic factors including age, marital status, chronic disease, medical insurance, endowment insurance, and monthly income per capita were included as covariates to control for potential confounding effects. The coefficient c’ represented the direct effect of social capital on depression. The indirect influence of social capital on depression through SPA was captured by the product of coefficients a and b (a × b). The total effect of social capital on depression was indicated by coefficient c, which is the sum of c’ and a × b. Point estimates were derived from 5,000 bootstrap samples, and 95% confidence intervals (CI) were calculated. An indirect effect was considered significant if the 95% CI did not include zero. All statistical tests were two-tailed, and the significance level for all analyses was set at 0.05. In addition, Cronbach’s alpha, ranging from 0 to 1, was used to assess the reliability of social capital scales. A Cronbach’s alpha of 0.6 or greater was considered acceptable (48).

## 3 Results

### 3.1 Sociodemographic characteristics and SDS score of samples

A total of 1706 community-dwelling older adult people over 65 years old were included in this study, with an average age of 73.75 ± 7.25. Most of them were female (56%), married (74.3%), had no chronic diseases (62.6%), had medical insurance (94.7%) and had endowment insurance (90.7%). Among all participants, the mean score of SDS was 39.07 (SD 13.97). The results of univariate analysis indicated that there were statistically significant differences in SDS score among older adults with different age, marital status, chronic disease, medical insurance, endowment insurance and monthly income per capita (*p* < 0.05). 80 years old and above, married, with chronic disease, with medical insurance, with endowment insurance, monthly income per capita 3,000 yuan and over, are the characteristics older adults with which have a lower level of SDS compared with other groups (Table 1).

**Table 1:** The status of SDS by different sociodemographic characteristics (*N* = 1706).

Variable	*n* (%)	SDS			
		Mean ± SD	*t* or *F*	*p* value	Post hoc
Age (years)			16.807	<0.001	
60–69^a^	597 (35.0)	37.00 ± 13.05			a < b,c
70–79^b^	691 (40.5)	39.01 ± 13.81			b < c
≥80^c^	418 (24.5)	42.11 ± 14.94			
Gender			0.352	0.725	
Male	751 (44.0)	38.93 ± 13.92			
Female	955 (56.0)	39.17 ± 14.01			
Marital status			4.108	<0.001	
Married	1,268 (74.3)	38.24 ± 13.81			
Divorce/Separate/Widowed	438 (25.7)	41.45 ± 15.16			
Education			1.925	0.124	
Primary school or below^a^	586 (34.3)	39.63 ± 14.41			
Junior high school^b^	596 (34.9)	39.45 ± 13.57			
Senior high school^c^	454 (26.6)	38.27 ± 14.13			
College or above^d^	70 (4.1)	36.23 ± 12.02			
Chronic disease			7.403	<0.001	
With	637 (37.3)	42.26 ± 14.84			
Without	1,069 (62.7)	37.16 ± 13.06			
Medical insurance			2.210	0.029	
With	1,616 (94.7)	38.89 ± 13.96			
Without	90 (5.3)	42.19 ± 13.76			
Endowment insurance			2.086	0.037	
With	1,548 (90.7)	38.84 ± 13.93			
Without	158 (9.3)	41.27 ± 14.23			
Monthly income per capita (RMB)			8.532	<0.001	
<1,000	265 (15.5)	41.27 ± 14.05			a > c,d
1,000–1,999	803 (47.1)	39.73 ± 14.31			b > c,d
2,000–2,999	497 (29.1)	38.09 ± 13.66			c > d
≥3,000	141 (8.3)	34.60 ± 11.62			

The lowercase a, b,c, and d represent different groups of the characteristics of different age, education and monthly income per capita.

### 3.2 Correlations between social capital, self-perceived aging and depression

Pearson’s r correlation analysis was conducted. The results indicated that social capital was significantly negatively correlated with depression (r = −0.263, *p* < 0.001) and SPA (r = −0.418, *p* < 0.001). Meanwhile, SPA was significantly positively correlated with depression (r = 0.324, *p* < 0.001) (Table 2). The significant correlation among the three variables indicates that their relationship can be further examined and elucidated through the development of a regression analysis model.

**Table 2:** Pearson correlation matrix of the variables of interest.

Variable	*r*			Mean ± SD
	1.	2.	3.	
1. Social capital	1	−0.418^***^	−0.263^***^	47.07 ± 6.72
2. Self-perceived aging	-	1	0.324^***^	45.33 ± 9.88
3. Depression	-	-	-	39.07 ± 13.97

*** *p*-value<0.001.

### 3.3 Mediation test for self-perceived aging

**Figure 2:**
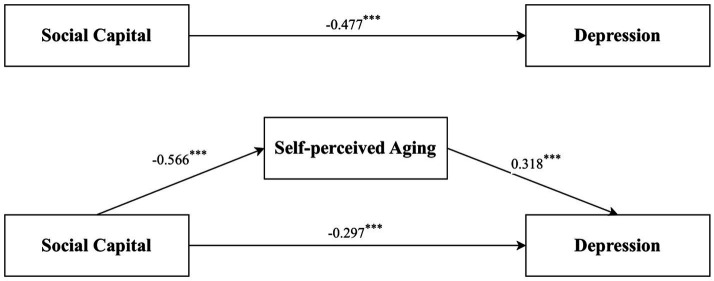
Self-perceived aging mediated the association of social capital and depression. ***p < 0.001.

Tables 3, 4 and Figure 2 presented the results of the mediation analysis conducted using the PROCESS macro. After adjusting for sociodemographic factors, the total effect of social capital on depression was significant (c = −0.477, *p* < 0.001). When SPA was introduced as a mediator, the direct predictive effect of social capital on depression persisted (c’ = −0.297, *p* < 0.001). In addition, social capital had a significant predictive effect on SPA (a = −0.566, *p* < 0.001), and the predictive effect of SPA on depression was also significant (b = 0.318, *p* < 0.001). Bootstrapping analysis with 5,000 samples confirmed a significant indirect effect (a × b = −0.180, 95% CI: −0.225 to −0.139), indicating that SPA acted as a mediator in the relationship between social capital and depression. Overall, the model explained 15.2% of the variance in depression.

**Table 3:** Multivariate linear regression analysis for the association among social capital, self-perceived aging and depression (*N* = 1706).

Variable	Model 1 (depression)			Model 2 (self-perceived aging)			Model 3 (depression)		
	B (SE)	*t*	*p* value	B (SE)	*t*	*p* value	B (SE)	*t*	*p* value
Age	0.184 (0.049)	3.769	<0.001	0.169 (0.033)	5.194	<0.001	0.130 (0.048)	2.707	0.007
Marital status	0.650 (0.806)	0.807	0.420	0.521 (0.538)	0.969	0.333	0.485 (0.788)	0.615	0.539
Chronic disease	4.345 (0.676)	6.424	<0.001	2.007 (0.451)	4.447	<0.001	3.706 (0.665)	5.574	<0.001
Medical insurance	−2.517 (1.828)	−1.377	0.169	0.648 (1.219)	0.532	0.595	−2.723 (1.787)	−1.524	0.128
Endowment insurance	1.004 (1.456)	0.689	0.491	−1.601 (0.972)	−1.648	0.010	1.513 (1.425)	1.062	0.288
Monthly income per capita	−1.988 (0.418)	−4.753	<0.001	−1.267 (0.279)	−4.543	<0.001	−1.585 (0.411)	−3.853	<0.001
Social capital	−0.477 (0.049)	−9.691	<0.001	−0.566 (0.033)	−17.227	<0.001	−0.297 (0.052)	−5.696	<0.001
Self-perceived aging							0.318 (0.036)	8.946	<0.001
*R* ^2^	0.112			0.210			0.152		
*F*	30.540 (*p* < 0.001)			64.342 (*p* < 0.001)			37.969 (*p* < 0.001)		

**Table 4:** Mediation analyses of self-perceived aging in the association between social capital and depression.

	Effect	95% bootstrap CI	Bootstrap SE
Total effect (c)	−0.477	(−0.573, −0.380)	0.049
Direct effect (c’)	−0.297	(−0.399, −0.195)	0.052
Indirect effect (a × b)	−0.180	(−0.225, −0.139)	0.022

## 4 Discussion

Against the backdrop of aging, this study takes the Chinese community-dwelling older adult as an example to explore and confirm the impact of social capital on depression in the older adult and the mediating role of SPA in the relationship between the two. This has important reference value for future theoretical and practical research aimed at improving depression among the older adult. Our study innovatively introduces SPA as a mediating mechanism, which has not been systematically examined in previous studies on social capital and depression among the older adult (23, 49). While prior research has emphasized the role of life satisfaction or social networks in mediating this relationship (50), our findings highlight the unique contribution of SPA in explaining how social capital shapes mental health outcomes.

Our study results show that there are significant differences in the SDS scores of the older adult in terms of age, marital status, chronic disease conditions, whether they have medical insurance, whether they have endowment insurance, and monthly income per capita, which is consistent with previous research findings (51–53). These identified non-modifiable factors can be used to determine which individuals are at risk of depression and to identify vulnerable groups that health professionals need to target. In health education, we should pay close attention to those aged 80 and above, who are divorced/separated/widowed, suffer from chronic diseases, without medical insurance, without endowment insurance, and have a monthly income per capita below 1,000 yuan, and provide more health intervention measures to improve their depressive symptoms.

This study found that social capital is negatively correlated with depressive symptoms among community-dwelling older adult individuals, confirming Hypothesis 1. A possible explanation for this correlation lies in the multifaceted nature of social capital, which encompasses trust, mutual aid, participation, and social connections. These elements of social capital serve as protective factors against depression by providing emotional support, information exchange, and practical assistance, which are crucial in alleviating stress and enhancing well-being (20, 54). The presence of a robust social network can act as a buffer against the negative impacts of stress and isolation, which are common among the older adult. When older adult individuals are embedded in supportive social networks, they are more likely to receive encouragement and validation, which can boost their self-esteem and sense of self-worth. This, in turn, can lead to a reduced likelihood of experiencing depressive symptoms. Furthermore, social capital can facilitate access to resources that are essential for maintaining mental health, such as healthcare services and community programs designed to promote well-being. Another aspect of social capital that may contribute to the observed negative correlation is the sense of belonging and community engagement. Active participation in community activities can provide a sense of purpose and belonging, which are known to be protective against depression (55). Older adult individuals who are actively engaged in their communities are more likely to experience positive social interactions, which can foster a sense of happiness and satisfaction with life, further mitigating the risk of depression (56, 57). Additionally, social capital can influence health behaviors and lifestyles (58, 59). Older adult individuals with higher social capital may be more likely to adopt healthy behaviors, such as regular physical activity and a balanced diet, which are known to have positive effects on mental health. They may also be more likely to seek help when needed, reducing the duration and severity of depressive episodes.

This study also found that SPA partially mediates the relationship between social capital and depression among community-dwelling older adult individuals, preliminarily clarifying the association mediation pathway between social capital and depression, and confirming Hypothesis 2. The mediating effect is mainly divided into two stages: (1) social capital has a negative impact on SPA, and (2) SPA has a positive impact on depression. The mediating effect of SPA in the relationship between social capital and depression can be explained in the following ways.

Notably, this mediation pathway extends prior work by identifying SPA as a critical psychological mechanism. While previous studies have linked social capital to depression through life satisfaction (49) or social networks (23), our findings suggest that social capital not only provides structural support but also shapes how individuals perceive their own aging process, which in turn affects mental health. This aligns with theoretical frameworks emphasizing the role of self-perception in aging outcomes (60).

Firstly, individuals with abundant social capital often receive more social support and positive social feedback, which helps them form a more positive SPA. Research has shown that social capital is associated with the mental health status and emotional depressive symptoms of the older adult. Positive social interactions and community participation can enhance the older adult’s sense of self-worth and social belonging, thereby reducing their negative perception of their own aging process (61). Secondly, a positive SPA can reduce the older adult’s feelings of loneliness and helplessness, which are important predictive factors for depressive symptoms (62). When older adult individuals hold a positive attitude towards their own aging, they are more likely to adopt a healthy lifestyle and participate in social activities, both of which help alleviate depressive symptoms.

Furthermore, the mediating role of SPA is also reflected in its ability to modulate the impact of social capital on depression. Specifically, individuals with higher social capital may receive more positive feedback due to stronger social connections and more frequent social interactions, which helps them to have a more positive view of aging (63). This positive view can reduce the occurrence of depressive emotions because it can enhance individuals’ coping abilities and life satisfaction. Conversely, individuals with lower social capital may have a more negative view of aging due to insufficient social support, which may increase their risk of depression.

Therefore, SPA, as a mediating variable, reveals the potential mediation pathway by which social capital may reduce the risk of depression by influencing older adult individuals’ perceptions of their own aging process. This finding provides important theoretical basis for the development of targeted mental health intervention measures in the future, especially in designing intervention projects aimed at increasing the social capital of the older adult and improving their positive views on aging. Through such interventions, we can expect to reduce the risk of depression in the older adult and improve their quality of life.

Moreover, our study contributes to the literature by providing empirical evidence from a rapidly aging society (China), where the older adult population has unique sociocultural contexts (e.g., family structures, healthcare access) compared to Western populations (64). This expands the generalizability of social capital theories in non-Western settings.

This study has some limitations. (1) First of all, this study is a cross-sectional study. Thus, we were unable to examine the causal relationship between social capital, SPA, and depression. For example, mental health status is also considered an important resource in later life. Older adults with good mental health can not only be more socially active, but they can also be more optimistic about their lives and tend to positively evaluate their social relationships and social resources. Future longitudinal studies with larger sample sizes and more variables are needed to address this important question. (2) The SDS Scale is a measure of self-reported depressive symptoms within the recent past week, which may lead to recall bias or be influenced by the current environment. (3) The study’s sample of community-dwelling older adult was drawn from a limited number of randomly selected communities within a single city, which could potentially introduce sampling bias and restrict the generalizability of the findings. Therefore, it is essential that future studies engage with community-dwelling older adult populations across various regions to enhance the applicability and breadth of the research outcomes.

## 5 Conclusion

Our research has identified a negative correlation between social capital and depression, with SPA acting as a partial mediator in this relationship. This suggests that enhancing social capital and fostering a positive SPA could be instrumental in preventing depression among older adults. This insight not only broadens the scope of social capital research but also offers a novel approach to addressing depression among Chinese community-dwelling older adult. It is recommended that relevant management agencies, communities, and families take effective measures to enhance the social capital of the older adult. Tailoring to their characteristics, strategies such as senior university programs, square dance clubs, senior chorus groups, and community sports events should be employed to motivate the older adult in building robust social networks. Additionally, fostering a positive outlook on aging is advised to improve their self-image and attitude towards the aging process.

## Data Availability

The original contributions presented in the study are included in the article/Supplementary material, further inquiries can be directed to the corresponding author.
