# Supplementary material for: The mediating effect of self-perceived aging on social capital and depression among Chinese community-dwelling older adult: a cross-sectional study
Source: Front Public Health. 2025 Jun 25;13:1571977. doi: 10.3389/fpubh.2025.1571977 (PMC12239832; doi:10.3389/fpubh.2025.1571977)
Supplement: Supplementary file 1 [file Table_1.docx]

Supplementary Material

# Supplementary Tables

Table S1. Coding of Variables

| Variable | Coding |
| --- | --- |
| Gender | Male = 1, Female = 2 |
| Marital status | Married = 1, Divorce/Separate/Widowed = 2 |
| Education | Primary school or below = 1, Junior high school = 2, Senior high school = 3, College or above = 4 |
| Chronic disease | With = 1, Without = 0 |
| Medical insurance | With = 1, Without = 0 |
| Endowment insurance | With = 1, Without = 0 |
| Monthly income (RMB) | <1000 = 1, 1000-1999 = 2, 2000-2999 = 3, >3000 = 4 |

Table S2. Descriptive analysis of Social Capital

| Variable | Total level score  (Mean ± SD) | Item score  (Mean ± SD) |
| --- | --- | --- |
| **Individual level** | 8.83 ± 2.23 |  |
| You have lots of close relationship in your life |  | 2.74 ± 1.04 |
| In the past month, you’ve usually been socializing with people except your family |  | 2.49 ± 1.11 |
| You always trust the people you associate with |  | 3.60 ± 0.79 |
| **Family level** | 14.89 ± 3.20 |  |
| You get on well with your children |  | 4.05 ± 1.03 |
| You get on well with your spouse |  | 3.48 ± 1.51 |
| Your family is always around you |  | 4.13 ± 0.99 |
| Your family always support you on financial in the past year |  | 3.24 ± 1.37 |
| **Association level** | 2.82 ± 1.77 |  |
| In the past year, you have usually participated in organization activities |  | 1.46 ± 1.01 |
| You have always received emotional or financial support from organization |  | 1.35 ± 0.85 |
| **Community level** | 9.55 ± 1.90 |  |
| If I have to move out of my present place, you will feel reluctant to do so |  | 3.03 ± 1.04 |
| You are very interested in what happens in your community: |  | 3.37 ± 0.77 |
| You always trust the people living in the same community |  | 3.15 ± 0.80 |
| **Society level** | 10.98 ± 1.96 |  |
| You have a lot of trust in medical institutions like hospitals and CDC |  | 3.64 ±0.80 |
| You have a lot of trust in government |  | 3.84 ± 0.85 |
| Hard work pays off in today’s society |  | 3.51 ± 0.81 |
| **Total Society Capital score** | 47.07 ± 6.72 |  |

Table S3. Descriptive analysis of Self-perceived Aging

| Variable | Total dimension score  (Mean ± SD) | Item score  (Mean ± SD) |
| --- | --- | --- |
| **Negative self-perceived aging** |  |  |
| Timeline-Chronic | 7.97 ± 2.93 |  |
| I always classify myself as old |  | 2.61 ± 1.13 |
| I am always aware of the fact that I am getting older |  | 2.69 ± 1.13 |
| I feel my age in everything that I do |  | 2.67 ± 1.13 |
| Consequences and Control Negative | 13.82 ± 4.66 |  |
| Getting older makes me less independent |  | 2.76 ± 1.14 |
| As I get older, I can take part on fewer activities |  | 2.81 ± 1.10 |
| As I get older, I do not cope as well with problems that arise |  | 2.97 ± 1.22 |
| Slowing down with age is not something I can control |  | 2.72 ± 1.10 |
| I have no control over the effects which getting older has on my social life |  | 2.56 ± 1.12 |
| Emotional Representations | 7.35 ± 2.26 |  |
| I get depressed when I think about how aging might affect the things that I can do |  | 2.61 ± 1.13 |
| I worry about the effects that getting older might have on my relationships with others |  | 2.51 ± 1.09 |
| I feel angry when I think about getting older |  | 2.23 ± 1.08 |
| **Positive self-perceived aging** |  |  |
| Consequences Positive | 7.54 ± 2.73 |  |
| As I get older, I get wiser |  | 2.10 ± 1.09 |
| As I get older, I continue to grow as a person |  | 2.61 ± 1.13 |
| As I get older, I appreciate things more |  | 2.83 ± 1.19 |
| Control Positive | 8.65 ± 3.11 |  |
| The quality of my social life in later years depends on me |  | 2.89 ± 1.23 |
| The quality of my relationships with others in later life depends on me |  | 3.01 ± 1.23 |
| Whether I continue living life to the full depends on me |  | 2.74 ± 1.14 |
| **Total Self-perceived Aging score** | 45.33 ± 9.88 |  |
